# Results of a pilot cluster randomised trial of the use of a Medication Review Tool for people taking antipsychotic medication

**DOI:** 10.1186/s12888-016-0921-7

**Published:** 2016-07-04

**Authors:** Joanna Moncrieff, Kiran Azam, Sonia Johnson, Louise Marston, Nicola Morant, Katherine Darton, Neil Wood

**Affiliations:** Division of Psychiatry, University College London, Maple House, 149 Tottenham Court Road, London, W1T 7NF UK; North East London Foundation Trust, Research & Development Department, Goodmayes Hospital, Barley Lane, Ilford, Essex IG3 8XJ UK; Department of Primary Care and Population Health and Priment Clinical Trials Unit, University College London, Rowland Hill Street, London, NE3 2PF UK; Mind, 15-19 Broadway, Stratford London, E15 4BQ UK; Goodmayes Hospital, Barley Lane, Ilford, Essex IG3 8XJ UK

**Keywords:** Antipsychotic drugs, Schizophrenia, Schizophrenia, Long-term treatment, Decision-making, Patient participation

## Abstract

**Background:**

Government policy encourages increasing involvement of patients in their long-term care. This paper describes the development and pilot evaluation of a ‘Medication Review Tool’ designed to assist people to participate more effectively in discussions about antipsychotic drug treatment.

**Methods:**

The Medication Review Tool developed consisted of a form to help patients identify pros and cons of their current antipsychotic treatment and any desired changes. It was associated with a website containing information and links about antipsychotics. For the trial, participants diagnosed with psychotic disorders were recruited from community mental health services. Cluster randomisation was used to allocate health professionals (care co-ordinators) and their associated patients to use of the Medication Review Tool or usual care. All participants had a medical consultation scheduled, and those in the intervention group completed the Medication Review Tool, with the help of their health professional prior to this, and took the completed Form into the consultation. Two follow-up interviews were conducted up to three months after the consultation. The principal outcome was the *Decision Self Efficacy Scale* (DSES). Qualitative feedback was collected from patients in the intervention group.

**Results:**

One hundred and thirty patients were screened, sixty patients were randomised, 51 completed the first follow-up assessment and 49 completed the second. Many patients were not randomised due to the timing of their consultation, and involvement of health professionals was inconsistent. There was no difference between the groups on the DSES (-4.16 95 % CI -9.81, 1.49), symptoms, side effects, antipsychotic doses or patient satisfaction. Scores on the Medication Adherence Questionnaire indicated an increase in participants’ reported inclination to adherence in the intervention group (coefficient adjusted for baseline values -0.44; 95 % CI -0.76, -0.11), and there was a small increase in positive attitudes to antipsychotic medication (Drug Attitude Inventory, adjusted coefficient 1.65; 95 % CI -0.09, 3.40). Qualitative feedback indicated patients valued the Tool for identifying both positive and negative aspects of drug treatment.

**Conclusions:**

The trial demonstrated the design was feasible, although challenges included service re-configurations and maintaining health professional involvement. Results may indicate a more intensive and sustained intervention is required to facilitate participation in decision-making for this group of patients.

**Trial registration:**

Current controlled trials ISRCTN12055530, Retrospectively registered 9/12/2013.

## Background

Providing more patient-centred care and facilitating self-management of medical treatment in long-term conditions is at the heart of the health agenda in the United Kingdom [1], along with much of the rest of the world. The “recovery movement” in mental health also aims to empower patients to build more fulfilling lives by exercising more control over their conditions and treatment [2, 3]. Following this agenda, many areas of medicine have introduced measures to help involve patients more closely in decisions about their treatment. A Cochrane review of trials of shared decision-making interventions indicated they could increase knowledge, increase confidence in decisions and facilitate more active patient involvement [4].

The importance of involving patients with mental health problems more closely in decisions about using psychiatric medication was highlighted by research which found high levels of non-adherence and extensive prescribing that exceeded recommended dose limits for antipsychotic drugs [5]. In response to these findings the United Kingdom government recommended that a more systematic consideration of the pros and cons of taking medication is needed and that patients should be more involved in medication reviews [6].

Antipsychotics effectively reduce acute psychotic symptoms and continuing use can prevent relapse [7, 8]. However, they are associated with a range of physical complications and are reported to be subjectively unpleasant to take [9, 10]. Hence levels of non-adherence are high [11]. Balancing the benefits of treatment against its potential negative effects is a complex task. If patients fail to appreciate the benefits of treatment and stop taking medication, they risk deterioration, relapse and hospitalisation [8]. On the other hand, patients report being more troubled by the adverse physical and mental effects of drug treatment than professionals commonly recognise [12] and these can significantly impair patients’ quality of life [13]. Reducing prescribed doses where possible may therefore improve adherence and reduce the personal and social costs associated with psychiatric morbidity, as well as those directly related to the physical complications of antipsychotics. One study found that 47 % of inpatients with schizophrenia were dissatisfied with decisions about medication, and this was more likely if they did not feel they had been involved in the decision [14].

National Institute for Health and Care Excellence (NICE) guidelines on the treatment of schizophrenia emphasise the importance of doctors and patients making collaborative decisions about drug treatment, based on “informed discussion” [15]. There has been little research on decision making in mental health, however, although people with schizophrenia and depression do express a desire to be more involved in decisions about their treatment [16, 17]. Some shared decision-making programmes for people with depression and schizophrenia indicated benefits in terms of knowledge levels, patient participation, satisfaction, improved adherence and symptoms [18–20]. However, the programmes for people with schizophrenia were conducted intensively with inpatients, and may prove difficult to translate into routine community mental health work. A recent internet-based decision aid aimed at people with schizophrenia in the community did not affect perceived involvement in decision-making [21]. Another community based intervention, using electronic care-planning to facilitate shared decision-making, improved satisfaction among health professionals (case managers) but not patients [22]. A recent systematic review of a diverse collection of eleven trials with a shared decision-making component, found a small impact on various measures of ‘empowerment’ (including measures of patient involvement and perceived efficacy in decision-making), and a trend towards reduced occurrence of future compulsory treatment. There was no overall effect on decision-making ability or quality of the therapeutic relationship [23].

### Aims and hypotheses

Many people attending community mental health services take antipsychotic drugs, often for long periods. The current research aimed to help patients assess and communicate more effectively about the personal risks and benefits that taking antipsychotic medication involves. By improving patient participation in the decision making process, we aimed to improve satisfaction with prescribed medication and clinical outcomes. We also aimed to improve prescribing practice, by focusing on patients’ main concerns, and encouraging the adjustment of antipsychotic doses where appropriate.

To facilitate these aims the first part of the research involved the development of a Medication Review Tool. The tool was designed for routine use in community mental health services, so we did not adopt the intensive methods of some previous shared decision making programmes. The Tool was intended to help patients systematically evaluate the pros and cons of their current antipsychotic medication, and to share their views with their psychiatrists. It was developed using insights from a qualitative study and refined and piloted in consultation with patients, carers and professionals. A pilot randomised controlled trial was then conducted to evaluate the use of the Medication Review Tool in community mental health services. The principal hypothesis was that use of the Tool would improve patients’ ability to participate effectively in decisions about their antipsychotic medication as measured by the *Decision Self Efficacy Scale* [24].

## Methods

### Intervention development

An initial draft of the Medication Review Tool was designed by the study management group, using insights from the literature on Decision Aids in general medicine. The Tool was modelled initially on the Ottowa Personal Decision Guide, and customised to address issues specific to antipsychotic medication [25]. It was designed to be accessible to both patients and professionals, to be implemented in routine practice, and to enable patients to consider all aspects of their antipsychotic treatment. Feedback on the first draft of the Tool was gathered during a qualitative interview study involving 20 participants with a diagnosis of schizophrenia, psychosis or schizoaffective disorder, who were taking antipsychotic medication (described fully elsewhere).

Following the study, a website was constructed, and a revised draft of the tool was incorporated into the website. Feedback on the website and the new draft of the Medication Review Tool was obtained through two group feedback sessions with patients (four participants each) and one with carers (five participants), and further individual consultation with two patients, one carer and two professionals.

The Medication Review Tool and website were finalised after the consultation. The website was designed to provide information about psychotic conditions including schizophrenia, types of antipsychotic medication and points for people to consider when discussing and making decisions about medication with professionals. It included links to external sites for users to access more detailed information. The Medication Review Tool, which was downloadable from the website, consists of a one page form, designed in an accessible format to allow patients to list the principal benefits and disadvantages of antipsychotic medication that they experience, changes they would like to be considered and other points for discussion. It is intended to be filled in by the patient, with support from a professional if necessary, and then taken into a psychiatric consultation about medication to enable them to express their views about medication more clearly and to have their concerns addressed more systematically. The website was password protected for the duration of the study to prevent contamination between groups during the pilot trial.

### Pilot trial

#### Participants

The study aimed to recruit a total of sixty (*n = 60*) participants from Community Recovery teams, which include patients with established mental health problems who need ongoing support, and Early Intervention in Psychosis Services, which cater for patients up to age 35 with a recent onset of psychotic illness. The study took place at the North East London Foundation Trust (NELFT) which covers a large section of outer London including economically deprived and more affluent areas. Participants had to be over the age of 18, have a diagnosis of psychosis, schizophrenia, schizoaffective disorder, delusional disorder or a mood disorder with psychotic symptoms and be currently taking antipsychotic medication. Participants were required to have an allocated health professional (care-coordinator or case manager), who was usually a nurse, social worker or occupational therapist from the participant’s clinical team. They also needed to have a consultation with their psychiatrist pending within the next three months, usually a Care Programme Approach meeting involving the psychiatrist (Patients on the ‘Care Programme Approach’ are assessed as having complex needs, have an allocated care-coordinator who acts as a keyworker and also coordinates all aspects of care, and are required to have regular multi-disciplinary reviews, which would normally involve a team psychiatrist).

Individuals who could not speak English or lacked capacity to consent were excluded from the study. The trial was approved by the Camden and Islington Research Ethics Committee, London (12/LO/0959).

#### Procedures

Health professionals who were willing to participate in the study were asked to approach potential participants from their caseload. The health professional then introduced the study and obtained verbal consent for the research team to contact the patient. Patients who agreed were sent an information sheet through the post, followed up by a telephone call by a member of the research team to provide more information and address any queries. If the patient agreed, a member of the research team then met with them to request informed consent and carry out the baseline interview.

**Fig. 1:**
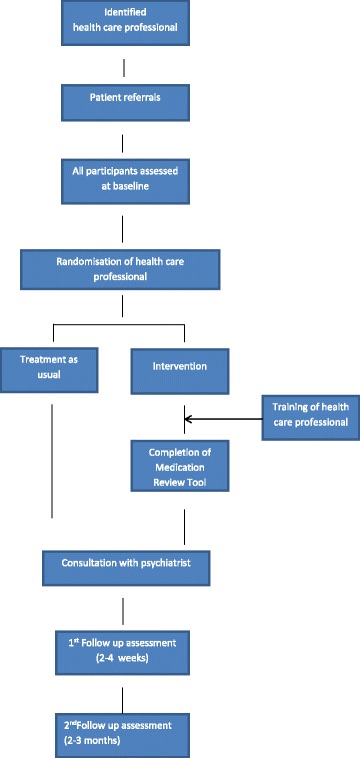
Study design

When all the potential study recruits associated with a particular health professional had completed their baseline assessments, the health professional was randomly allocated either to use the Medication Review Tool with participants, or to provide ‘treatment as usual’ (Fig. 1). Health professionals were intended to be involved in helping patients engage with the intervention due to chronicity of symptoms and high levels of functional impairment in the study population. Health professionals allocated to the intervention group were provided with 15 minute training session on how to help patients use the Medication Review Tool. Training consisted of explaining the rationale of the study, introducing health professionals to the website and the Form, illustrating how they could help participants access further information and consider a range of medication-related issues, and demonstrating how they would complete the Form with participants.

Thus the study was a cluster randomised trial with health professionals as the unit of randomisation. It was not possible to blind patients or health professionals due to the nature of the intervention, and data collection was not blinded due to the fact there was only one principal researcher assigned to the study. Statistical analyses were conducted blind, however (see below).

Cluster randomisation was carried out using an internet based randomisation service (Sealed Envelope) using blocks of size 4, 6 and 8. The allocation list was held by an independent administrator from Priment clinical trials unit at University College London in order to achieve concealment of allocation. Following randomisation, participants in the intervention group were sent a link to the website along with the login details (username and password). Individuals who did not have access to the internet were sent printed hard copies of the information on the website. The website details or information was sent to the participant a few weeks before the pre-booked consultation so that they could have the opportunity to look through the website and the form in their own time if they chose to do so prior to looking at it with their health professional.

Participants then met with their key health professionals or care-coordinators to look at the website and complete the form. This meeting was intended to take place about a week before the consultation. When clients had completed the form, a copy was placed in their electronic clinical records, and they were given the hard copy to keep and take to the meeting. Consultations with psychiatrists normally involve a review of the patient’s medication and the completed form was intended to be shared with the psychiatrist during the meeting in order to contribute to this process.

#### Outcome assessment

Follow-up was conducted in two stages. There was an initial telephone or face to face interview conducted 2 to 4 weeks after the participant’s consultation meeting. This interview focused on feedback and measures of the nature of the consultation. The second follow-up interview took place face to face two to three months after the consultation, and focused on symptoms, side effects and adherence (Fig. 1).

The primary outcome measure was the *Decision Self-Efficacy Scale (DSES),* an 11-item questionnaire used to assess participants’ confidence in participating in clinical discussions and decisions [24]. Each item has five possible responses, which are scored 0 (not at all confident), to 4 (very confident). These scores are summed giving a total score between 0 and 44, with higher scores indicating higher decision self-efficacy. This measure has been validated in people with schizophrenia [26]. Participants completed the questionnaire at baseline and at the first follow-up.

Secondary outcomes included total scores on the *Client Satisfaction Questionnaire (CSQ-8)* [27], collected at first follow-up, and the *Drug Attitude Inventory 10 (DAI-10)* [28], the *Liverpool University Neuroleptic Side Effect Rating Scale (LUNSERS)* [29] *and* the *Brief Positive and Negative Syndrome Scale (Brief PANSS)*, all collected at second follow-up. The *Brief PANSS* was developed by Yamamoto et al 2010 and correlates highly with the full PANSS [30]. We added an item on hallucinations, since this is not included, and it was anticipated to be a common symptom among our sample. At second follow-up the *Medication Adherence Questionnaire* [31] was also completed, and an item from the Tablets Routine Questionnaire [32] on doses of medication missed during the preceding week. Participants were also asked about any changes to their antipsychotic and other medications since the baseline interview, and these data were checked against medical records, with the participant’s permission. Daily doses of antipsychotics in chlorpromazine equivalents were calculated at baseline and follow-up, and the number of different antipsychotic drugs patients were taking was also recorded.

Feedback on the implementation of the intervention, and qualitative data concerning participants’ views on the process of using the Medication Review Tool was collected using a short semi-structured interview at the first follow-up point with participants allocated to this group. This included questions about the practicalities of using the tool and website, how the tool had been employed during the consultation, and whether the participant had found the process helpful or not. Responses were either audio-recorded or recorded in note form by the interviewer.

#### Data analysis

Quantitative data was entered into the Statistical Package for the Social Sciences (SPSS) [33], and then transferred into Stata version 13 for statistical analysis [34]. Demographics and baseline variables were described. A formal sample size calculation was not carried out due to the study being a pilot, but we aimed to recruit 60 service users. This number was thought to be more than adequate to assess trial processes and obtain outcome data with which to perform future sample size estimation.

Continuous and dichotomous outcomes were analysed using random effects modelling, to account for clustering by care coordinator. All models included only the baseline value of the outcome (where appropriate) and the randomised group. This is because of the relatively small sample size not supporting further variables in the model and the decreased likelihood of the model converging with more variables included.

**Fig. 2:**
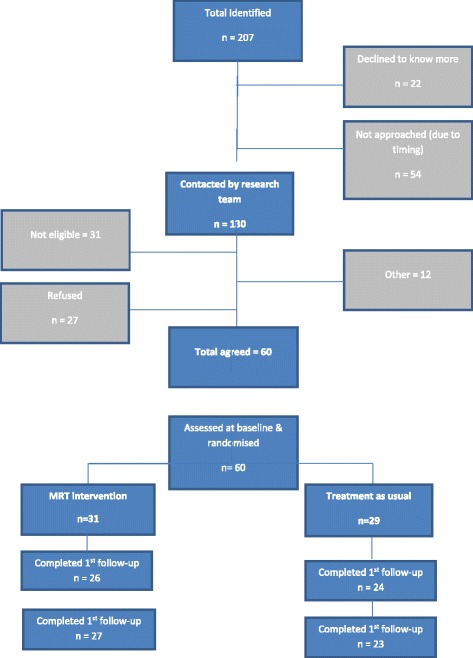
Recruitment flowchart (CONSORT diagram)

Process outcomes included recruitment rate, dropout rate and number (percentage) of completers. These are reported using the Consolidated Standards of Reporting Trials (CONSORT) diagram (Fig. 2). The intraclass correlation coefficient was calculated for the primary outcome (DSES) using the health professional as the cluster. All statistical analyses were conducted by the study statistician who remained blind to allocation until all the results were agreed.

Qualitative feedback data were analysed using a theoretically-oriented form of thematic analysis [35], that focused on two issues: descriptions of how the Medication Review Tool had been implemented, and experiences of the impact of using the tool. Both commonalities and variations in respondents’ accounts of implementation process and the interventions’ impact were explored.

## Results

### Process outcomes

The CONSORT diagram for the trial is presented in Fig. 2. Sixty participants were recruited over a period of 11 months. Thirty-one participants were randomised to the intervention group and 29 to the treatment as usual group. A total of 54 potential participants (26 %) out of the 207 individuals referred for the study were not able to be included in the study because it was not possible to organise a baseline assessment prior to the clinical consultation. Thirty one individuals were ineligible (in most cases due to not having a diagnosis of a psychotic disorder), 49 declined participation and 12 were not included for other reasons such as the participant or care-coordinator not responding to contact requests. The cluster design required that all the patients assigned to a particular health professional had to have baseline interviews before the care-coordinator could be randomised. Delays in organising baseline interviews for some participants meant pre-scheduled psychiatric consultations came and went before they were randomised. In order to keep participants who had already had baseline assessments in the study therefore, sometimes a decision had to be taken to randomise health professionals before all their potential patients had had the opportunity to have a baseline interview. Reasons for delay in organising baseline assessments included difficulty in contacting participants, participants moving house, being admitted to hospital, or having other less severe deteriorations of mental state, and delay in health professionals contacting patients to make the initial introduction to the research.

Twenty seven of those randomised to the intervention group completed the intervention. Implementing the Medication Review Tool intervention was also difficult partly due to service reconfigurations that occurred during the course of the study, which meant there were frequent changes to participants’ allocated health professionals. Some health professionals also found it difficult to find time to do the training or complete the form with clients, and in five cases the research assistant performed this task instead due to care-coordinators being ill or unavailable for other reasons.

Rates of follow-up were good, with 85 % of patients completing the first follow-up, and 81 % the final follow-up. Qualitative data was obtained from 22 patients in the intervention group.

### Participant characteristics

Demographic characteristics and baseline values of outcome measures are displayed in Table 1. Those in the intervention group were on average six years older than those in the intervention group and a greater percentage were single, unemployed and white. Antipsychotic use was comparable between groups, as was other medication; though a greater percentage in the treatment as usual group were taking anticholinergic drugs and drugs referred to as ‘mood stabilisers’. Baseline values for outcome measures were similar between groups (Table 2).

**Table 1:** Baseline characteristics

Variable	Treatment as usual		Medication review tool	
	n/N or mean	% or (SD)	n/N or mean	% or (SD)
Male	20/29	69	23/31	74
Age	39	(11)	45	(10)
White	21/29	72	25/31	81
Single	20/29	69	25/31	81
Unemployed	20/29	69	24/31	77
Number of years been in contact with mental health services				
Less than a year	1/29	3	1/31	3
1 to 3 years	3/29	10	4/31	13
4–10 years	5/29	17	2/31	6
More than 10 years	20/29	69	24/31	77
One antipsychotic	22/29	76	27/31	87
Two antipsychotics or more	7/29	24	4/31	13
Total dose per day (Chlorpromazine equivalent (mg)) median (IQR)	400	200, 600	250	132, 400
Taking other medication	24/29	83	23/31	74
Antidepressants	9/29	31	10/31	32
Anxiolytic	2/29	7	3/31	10
Anti cholinergic	10/29	34	7/31	23
Mood stabilisers	9/29	31	5/31	16
Medication for physical illnesses	17/29	59	18/31	58

**Table 2:** Analysis of outcomes

	Treatment as usual		Medication review tool		Coefficient or OR (95 % CI)
	Baseline	Follow-up	Baseline	Follow-up	
Outcome					
Decision Self Efficacy Scale (mean, SD)	29 (9)	32 (10)	29 (10)	30 (9)	-4.16 (-9.81, 1.49)
Drug Attitude Inventory (mean, SD)	5 (4)	3 (4)	5 (4)	5 (3)	1.65 (-0.09, 3.40)
LUNSERS (mean, SD)	38 (23)	28 (19)	38 (23)	30 (20)	-0.42 (-8.12, 7.29)
Brief PANSS (mean, SD)	18 (7)	16 (7)	17 (7)	16 (6)	0.13 (-2.21, 2.48)
Client Satisfaction Questionnaire (mean, SD)		28 (5)		27 (5)	-0.29 (-3.04, 2.45)^a^
Medication Adherence Questionnaire (median, IQR)	0 (0, 1)	1 (0, 1)	0 (0, 0)	0 (0, 1)	-0.44 (-0.76, -0.11)
2 or more antipsychotics taken (n/N, %)	7/29 (24)	7/24 (29)	4/31 (13)	7/26 (27)	0.89 (0.26, 3.08)^b^
Dose (chlorpromazine equivalents) (median, IQR)	400 (200, 600)	350 (200, 648)	250 (132, 400)	292 (200, 462)	-26.02 (-97.23, 45.20)
Change in chlorpromazine equivalent baseline-FU (median, IQR)		0 (0, 50)		0 (-64, 65)	3.33 (-78.12, 84.79)

*Abbreviations*: *SD* standard deviation, *LUNSERS* Liverpool University neuroleptic side effect rating scale, *PANSS* positive and negative syndrome scale, *IQR* interquartile range, *FU* follow-up, *OR* odds ratio

^a^There is no baseline adjustment for this analysis

^b^Odds ratio. Data were too sparse to do the adjusted analysis

### Efficacy

Table 2 shows the baseline and follow-up values of the measures used, with comparisons between groups adjusted for baseline values where possible. Contrary to the study hypothesis, the Decision Self Efficacy Scale scores were lower in the Medication Review Tool group than in the Treatment as usual group after adjustment for baseline score -4.16 (95 % CI -9.81, 1.49). Scores on the Medication Adherence Questionnaire indicated a statistically significant difference with patients in the intervention group indicating a greater tendency to be adherent with medication compared to those in the treatment as usual group. The Medication Adherence Questionnaire consists of four questions about participants’ inclination to adhere to medication, such as whether they ever forget to take medication, or are sometimes careless about taking it [31]. Higher scores indicate less inclination to adherence. Attitudes towards antipsychotic treatment as measured by the Drug Attitude Inventory [28] were also more favourable in the intervention group (higher scores indicate better subjective response to medication) (Table 2).

There were no differences in brief PANSS scores, side effects, doses of antipsychotics at follow-up in chlorpromazine equivalents, use of two or more antipsychotics or in client satisfaction at follow-up (Table 2).

More patients in the intervention group had changes in their antipsychotic medication. Nine had an increase in dose as measured by total chlorpromazine equivalents, and eight had a decrease (*N* = 25). In the treatment as usual group six had an increase, and four had a decrease *(N* = 24). Overall, however, there was no difference between the groups in terms of the change in the chlorpromazine equivalent dose of antipsychotics (Table 2).

The intraclass correlation coefficient (ICC) for the primary outcome, the *DSES*, for clusters greater than one, was 0.07 (95 % CI 0.00, 0.46).

### Implementation feedback

Six of the 22 respondents reported completing the Medication Review Tool with the care-coordinator as planned, but nine reported that they had completed it on their own, and two said they did it with the psychiatrist during their consultation. Three could not remember completing the Tool (although they had done so) and two did not provide any information on this. Respondents reported spending between two minutes and 30 min considering and completing the Medication Review Tool, the median time being 10 min (based on data from 18 respondents). Fifteen of the 22 respondents recalled showing the Tool or form to their psychiatrist during the consultation, or in one case discussed the contents of the form during the consultation, but completed it afterwards. Two respondents had not shown the Tool to their psychiatrist, and were not certain if the psychiatrist had seen a completed copy or not. One of these respondents was disappointed; the other was not concerned since there had been a satisfactory discussion of medication during the consultation in any case.

When asked about their use of the supporting information, six respondents had read the printout of the information on the website which had been sent to them. Only one had looked at the website itself, however. Nine had looked at neither and five did not supply any information. Three of those who looked at the information on the website or in printed form described it as helpful, three said it was not helpful, and one did not express an opinion.

### Impact of using the medication review tool

Most respondents reported finding the Medication Review Tool helpful to some degree (*N* = 13). Reasons given were that completing the Medication Review Tool helped to clarify the benefits of antipsychotic medication, or the reasons why someone might be taking it, and in some cases its side effects.

Two participants suggested it had resulted in a more positive attitude towards medication:

‘It made me understand why I am taking what I am taking’ (P 27).

‘I was able to identify what was good about the medication that I hadn’t done before. I always used to think about the negatives. It made me feel better about the medication doing some good’ (P 44).

Four participants suggested that completing the form had made them aware of the full extent of the side effects they experienced.

[completing the Tool] ‘made me think about all the side-effects of the medication and realise that, you know, I needed to push forward about getting something done about it’ (P 33).

Five participants felt that completing the form had influenced the way clinical staff viewed their medication, and in some cases had led to adjustments in doses of medication during the medication review meeting:

‘it made her [the psychiatrist] see all the side effects and what they do’ (P 19).

One participant was disappointed that the psychiatrist had not taken any notice of the form and its contents, and two others felt it had made no difference to their own views about medication, or to the outcome of their consultation.

Two participants said they had no interest in the process and had not found it helpful. One of these described how completing the Medication Review Tool had made him anxious, because he became generally anxious when having to complete forms.

## Discussion

### Process outcomes

The study showed that it was possible to introduce a Medication Review Tool designed to improve patients’ ability to participate in discussions and decisions about their antipsychotic medication into routine clinical services. The pilot trial demonstrated that patients could be recruited, randomised and most retained for follow-up at three months. The intervention was not always implemented as planned, however, and the involvement of health professionals (care-coordinators) was inconsistent. Feedback illustrated that most participants did not access information on the internet, and were more likely to read printed information. The fact that the website was password protected might have acted as a deterrent to some participants.

### Efficacy results

Although this was not a fully powered trial, the results did not suggest that the use of a Medication Review Tool for patients with psychotic disorders and schizophrenia increased participants’ confidence in making decisions about their antipsychotic treatment. It may have increased the inclination towards medication adherence and produced marginally more positive attitudes towards antipsychotic medication. Given the small sample size these findings could be false positives, but alternatively they may indicate that providing patients with the opportunity to reflect on their treatment made them more aware of its positive effects. This was supported by qualitative data from some patients, although others suggested that the process highlighted the negative aspects of medication. In contrast, a German study of shared decision-making training for people with schizophrenia found that patients who underwent training became more sceptical about medication [20]. Another possibility is that use of the Tool could lead to dose reduction which might enhance positive attitudes to medication by reducing side effects. However, there was no evidence that dose reduction had occurred more commonly among the intervention group in this study. Alternatively, the present finding may reflect a Hawthorne-type effect, whereby the process of using the Medication Review Tool made participants more inclined to make positive statements about medication to gain the interviewer’s approval.

The intervention did not affect symptoms of schizophrenia, side effects or dosages of antipsychotics. The qualitative feedback indicated that some patients found using the Medication Review Tool helped them clarify benefits and side effects of antipsychotic medication, and to think about what changes, if any, they would like to be made to their medication in the future. Several patients felt that using the form had influenced clinical staff and given them more understanding of the patient’s experience of taking these particular drugs. Others, however, had little interest in considering their medication in more detail, and some forgot they had used the instrument at all.

### Limitations

The design of the study was challenging in several ways. Firstly, although cluster randomisation helps prevent contamination, the current design meant many willing and eligible patients could not be included, because of the need to complete baseline interviews on all the patients allocated to a health professional prior to randomisation. This problem was greatly exacerbated by service re-configurations and changes to staff that took place during the course of the study. Individual randomisation may have been easier to implement, but would have risked contamination either through participants comparing treatment or by care coordinators introducing the intervention, or elements of it, to participants in the treatment as usual group. Cluster randomisation by team would have avoided the problem of contamination, but requires larger sample sizes, and would probably have taken longer to recruit all potential participants from a team before randomisation could take place. A non-randomised cohort study comparing outcomes of patients under the care of teams in which the Medication Review Tool was introduced with patients from other, comparable teams would have been easier to implement, but would have had lower credibility than a randomised trial, since there are likely to be differences between groups due to geographical location, demographics and illness profile.

Secondly, the participation of health professionals was inconsistent, due to competing demands on their time. The present study was aimed at enhancing patients’ ability to communicate their wishes about medication, but the lack of attention paid to psychiatrists, and their part in discussing treatment options, may have limited the implementation and efficacy of the intervention.

Thirdly, the study population may have found it difficult to engage with a short and simple intervention such as the one developed. Although some participants reported finding the Medication Review Tool useful, others did not, and some had even forgotten using it. This may reflect the extent to which some people with long histories of mental illness come to feel disempowered and disengaged, perhaps through a combination of the nature of their condition and experiences of services. A one-off intervention, such as the one investigated here, may be insufficient to improve patient involvement in decision-making for this population. A recent trial of an internet-based shared decision making programme for community patients with psychotic disorders in the Netherlands also found that it failed to produce the anticipated improvements in participation in decision-making. Moreover, only a third of patients in the intervention group accessed the web-based decision aid that was provided to them [21]. A cross-diagnostic electronic shared-decision making intervention evaluated in community mental health services in the United States also failed to improve patient satisfaction, although it did increase patient recall of their care plans [22].

Other limitations include the fact that the trial was only conducted with English-speaking patients, which restricts its generalisability to non-English speaking populations, who are likely to have particular difficulties with communicating views about their treatment.

### Implications for further research

Qualitative work would be useful to explore the obstacles to further involvement of patients with long-term mental illness in decisions about their treatment. For people who have been involved with services for many years, more intensive, repeated interventions, such as those trialled in inpatient settings, may be required [19, 20]. Education aimed at professionals, teams and services may also be necessary to instil a culture in which more active patient involvement is accepted as worth promoting. A design using whole teams as clusters could be more effective than the current design, with a sustained and repeated intervention integrated into team working practices, and education and training conducted at a team level. Further involvement of psychiatrists in the process of enhancing decision making could also improve levels of patient participation. Psychiatrists were not directly involved in administering the current intervention, and including training for psychiatrists to facilitate patient involvement might improve chances of success.

## Conclusions

The study set out to develop a tool to help patients become more involved in making decisions about the use of antipsychotic medication and to test this tool in a pilot trial. The trial was implemented successfully, but process data pointed to difficulties arising from timing pressures, staff motivation and service reconfigurations. Although not powered to provide definitive data, the results did not suggest that the tool had the intended impact in patients’ confidence in decisions about their medication. It may have had a small effect on attitudes towards adherence and to antipsychotic medication generally, although these may be chance findings, given the small numbers. In qualitative feedback, patients identified the benefits of using an instrument that allowed them to focus on the positive and negative aspects of antipsychotic treatment, and to communicate their experiences to clinical staff. The research may reflect the ongoing difficulties of introducing new priorities, and the challenges that still lie ahead for efforts to empower long-term users of mental health services.

## Abbreviations

CI, confidence interval; CONSORT, consolidated standards of reporting trials; CSQ-8, client satisfaction questionnaire, version 8; DAI 10, drug attitude inventory, 10 item version; DSES, decision self efficacy scale; FU, follow-up; ICC, intraclass correlation coefficient; IQR, interquartile range; LUNSERS, Liverpool University neuroleptic side effect rating scale; NELFT, North East London Foundation Trust; OR, odds ratio; PANSS, positive and negative syndrome scale; SPSS, statistical package for the social sciences
